# Different Effects of High-Fat/High-Sucrose and High-Fructose Diets on Advanced Glycation End-Product Accumulation and on Mitochondrial Involvement in Heart and Skeletal Muscle in Mice

**DOI:** 10.3390/nu15234874

**Published:** 2023-11-22

**Authors:** Eleonora Aimaretti, Guglielmina Chimienti, Chiara Rubeo, Rosa Di Lorenzo, Lucia Trisolini, Federica Dal Bello, Atefeh Moradi, Massimo Collino, Angela Maria Serena Lezza, Manuela Aragno, Vito Pesce

**Affiliations:** 1 Unit of Experimental Medicine & Clinical Pathology, Department of Clinical and Biological Science, University of Turin, 10125 Turin, Italy; eleonora.aimaretti@unito.it (E.A.); chiara.rubeo@unito.it (C.R.); atefeh.moradi@edu.unito.it (A.M.); manuela.aragno@unito.it (M.A.); 2 Department of Biosciences Biotechnologies and Environment, University of Bari Aldo Moro, Via Orabona 4, 70125 Bari, Italy; guglielminaalessandra.chimienti@uniba.it (G.C.); rosa.dilorenzo@uniba.it (R.D.L.); 3 Institute of Biomembranes, Bioenergetics and Molecular Biotechnologies, National Research Council, CNR, 70125 Bari, Italy; l.trisolini@ibiom.cnr.it; 4 Department of Molecular Biotechnology and Health Sciences, University of Turin, 10125 Turin, Italy; federica.dalbello@unito.it; 5 Department of Neuroscience “Rita Levi Montalcini”, University of Turin, 10125 Turin, Italy; massimo.collino@unito.it

**Keywords:** high-fat high-sucrose diet (HFHS), high-fructose diet (HFr), inflammation, advanced glycation end-products (AGEs), mitochondrial oxidative stress, mitochondrial biogenesis, mtDNA maintenance, mitochondrial dynamics

## Abstract

Diets with an elevated content of fat, sucrose, or fructose are recognized models of diet-induced metabolic alterations, since they induce metabolic derangements, oxidative stress, and chronic low-grade inflammation associated with local and systemic accumulation of advanced glycation end-products (AGEs). This study used four-week-old C57BL/6 male mice, randomly assigned to three experimental dietary regimens: standard diet (SD), high-fat high-sucrose diet (HFHS), or high fructose diet (HFr), administered for 12 weeks. Plasma, heart, and tibialis anterior (TA) skeletal muscle were assayed for markers of metabolic conditions, inflammation, presence of AGEs, and mitochondrial involvement. The HFHS diet induced a tissue-specific differential response featuring (1) a remarkable adaptation of the heart to HFHS-induced heavy oxidative stress, demonstrated by an increased presence of AGEs and reduced mitochondrial biogenesis, and efficaciously counteracted by a conspicuous increase in mitochondrial fission and PRXIII expression; (2) the absence of TA adaptation to HFHS, revealed by a heavy reduction in mitochondrial biogenesis, not counteracted by an increase in fission and PRXIII expression. HFr-induced mild oxidative stress elicited tissue-specific responses, featuring (1) a decrease in mitochondrial biogenesis in the heart, likely counteracted by a tendency for increased fission and (2) a mild reduction in mitochondrial biogenesis in TA, likely counteracted by a tendency for increased fusion, showing the adaptability of both tissues to the diet.

## 1. Introduction

The consumption of energy-rich diets with elevated content of fats and sugars (i.e., ‘Western style’ or ‘junk food’ diet), combined with a sedentary lifestyle, may induce a positive energy balance responsible for weight gain and obesity. Obesity is a serious global public health concern nowadays since it promotes the development of metabolic syndrome (MS), type 2 diabetes (T2D), non-alcoholic fatty liver disease (NAFLD), and cardiovascular disease (CVD) [1,2,3]. Moreover, obesity is also characterized by a chronic low-grade inflammatory state in peripheral tissues [4] and an overproduction of reactive oxygen species (ROS) [5].

Recent studies have highlighted the effects of specific nutrients on insulin sensitivity and fat storage [6]. Fructose, among others, plays a notable role: indeed, its intake has heavily increased in the last decades due to changes in dietary behaviors, such as higher consumption of sugar-sweetened beverages and sugar-rich processed foods [7]. Clinical trials and experimental studies suggest that a high fructose intake is an important causative factor of metabolic derangements [8] associated with an excessive inflammatory response and oxidative stress [9,10]. 

In the pathogenesis of chronic metabolic diseases characterized by insulin resistance, mitochondrial dysfunction has been implicated as a major feature, although mitochondrial dysfunction may also occur independently of hyperglycemia, arising from lipotoxicity, oxidative stress [11], or inflammatory conditions [12,13]. In particular, the mitochondria, which are the major cell source of ROS through respiratory chain complexes, enhance ROS production because of the HFHS-diet-related increased metabolic utilization of fatty acids through β-oxidation, leading to oxidative stress conditions [14]. 

Additional support to diet-induced oxidative stress and inflammation comes from the presence of advanced glycation end-products (AGEs), which are a group of heterogeneous compounds. AGE levels in vivo derive not only from endogenous formation, but also from exogenous sources [15]. Glucose reacts with free amino groups (in lysine or arginine), producing Schiff bases which, after a series of complex reactions, generate AGEs, specifically Nε-(carboxymethyl)-lysine (CML), pentosidine, furosine, GOLD (6-{1-[(5S)-5-ammonio-6-oxido-6-oxohexyl]imidazolium-3-yl}-L-norleucine/GlyOxylderived Lysine Dimer), and MOLD (6-{1-[(5S)-5-ammonio-6-oxido-6-oxohexyl]-4-methyl-imidazoli/MethylglyOxal-Lysine Dimer) [16]. AGEs’ toxicity is exerted by the cross-linking of proteins, leading to alterations in tissue/vessel structure and by their interaction with cell surface receptors, including RAGE (receptor for advanced glycation end-products), one of the most investigated AGE receptors, belonging to the immunoglobulin (Ig) superfamily [17]. The resulting carbonyl molecules accumulate in the body and are very reactive and cause carbonyl stress, which in turn can aggravate inflammation and oxidative stress. Therefore, the administration of a high-fat high-sucrose (HFHS) diet or of a high-fructose (HFr) diet in vivo allows to mimic the effects of dangerous feeding habits both at the level of specific organs and of the whole organism [18]. Our interest was aroused by the possible consequences of the exposition to a HFHS or HFr diet in tissues particularly challenged by mitochondrial oxidative stress, such as the heart and skeletal muscle. A comparison between these tissues led us to scrutinize their specific capacity to adapt to the oxidative stress imposed by diets and to draw conclusions that are also relevant at a translational level. In fact, very few papers have examined the heart and skeletal muscle of HFHS-diet-treated rodents while at the same time [19,20,21,22] focusing on the impact of diet-induced metabolic derangements on the exacerbation of myocardial and muscle damage. The present study aimed to (1) compare two very different diets in terms of calories intake and source of calories (HFHS vs. standard diet, SD), for their respective effects on the level of AGE formation and on mitochondrial functions (biogenesis, dynamics and oxidative stress) in the heart and tibialis anterior (TA) skeletal muscle; (2) compare the above-indicated effects between a diet isocaloric with SD, in which the carbohydrates are derived almost completely from fructose, and SD (HFr vs. SD), to evaluate the possible consequences of the nowadays enormously increased fructose intake. The detection of potential differences between the responses induced by a HFHS or HFr diet may add a translational value to the work, allowing us to characterize tissue-specific alterations and to extend our investigation from mitochondrial dysfunction, deeply analyzed at the biogenesis, dynamics, and oxidative stress levels, to the skeletal muscle and heart metabolic contexts.

## 2. Materials and Methods

### 2.1. Animals

This study was conducted in forty-five 3-week-old C57BL/6JOlaHsd male mice (Envigo RMS Srl, Udine, Italy). The animals were housed in cages of 5 mice each and kept under conventional laboratory conditions, at 25 ± 2 °C and under an automatically controlled light/dark cycle (12/12 h). After 1 week of acclimatization, mice were randomly assigned to the following dietary regimens: standard diet (SD, *n* = 15), high-fat high-sucrose diet (HFHS, *n* = 15), and high-fructose diet (HFr, *n* = 15) for 12 weeks. The standard diet (3.85 kcal/g) provided 10% kcal from fat, 70% kcal from complex carbohydrates (corn starch, maltodextrins) and 20% kcal from protein; the HFHS diet (5.56 kcal/g) supplied 58% kcal from fat (coconut oil, hydrogenated), 26% kcal from carbohydrates (sucrose, maltodextrins) and 16% kcal from protein. Finally, the HFr diet, which was isocaloric with the SD diet, provided 10% kcal from fat, 70% kcal from carbohydrates, of which 60% were from fructose and 10% from corn starch, and 20% kcal from protein. Food pellets were ordered from Research Diet (New Brunswick, NJ, USA) and the codes were, respectively, D12450K, D12331, and D02022704.

At the end of the in vivo protocol, animals were anesthetized with isoflurane (IsoFlo, Abbott Laboratories, North Chicago, IL, USA) and euthanized by cardiac puncture and exsanguination. Blood samples were collected in microtubes containing EDTA and then centrifuged at 10,000× *g* at room temperature to separate plasma, which was directly snap-frozen. Samples of the heart (right ventricle, which is the part most sensitive to oxidative stress [23]) and skeletal muscle (tibialis anterior) from each mouse were weighted, collected in tubes, and frozen in liquid nitrogen. Samples were then stored at −80 °C until further analyses.

### 2.2. Oral Glucose Tolerance Test (OGTT)

After 12 weeks, an OGTT was performed following a 12 h fasting period. After the measurement of basal fasting blood glucose, 2 g/kg of glucose were administered (30% aqueous solution, Sigma Aldrich, St. Louis, MO, USA) by oral gavage and serum glucose was measured by tail puncture after 15, 30, 60, and 120 min with a conventional glucometer (OGTT, Accu-check Aviva, Roche Diabetes Care Italy S.p.A, Monza, Italy).

### 2.3. Insulin Tolerance Test (ITT)

At the end of the 12-week protocol, following a 6 h fasting period, an ITT was performed. Briefly, glucose at the basal level was measured and, right after, insulin was administered intraperitoneally at a dose of 0.75 IU of insulin/kg of body mass. Glycemia was then evaluated after 15, 30, 60, and 90 min with a glucometer (Accu-check Aviva, Roche Diabetes Care Italy S.p.A, Italy).

### 2.4. Evaluation of Biochemical Markers

After terminal procedures, plasma levels of the following markers were measured using commercially available clinical assay kits (FAR Diagnostics, Verona, Italy) according to the manufacturer’s instructions: cholesterol (kit n° 7050), liquid triglycerides (kit n° 7136), LDH (lactate dehydrogenase, kit n° 7096). Interleukin-6 (IL-6) was measured with an enzyme-linked immunosorbent assay (ELISA assay) from Abcam (Cambridge, UK).

### 2.5. Nε-(Carboxymethyl)-Lysine (CML) Levels in Plasma and Tissues 

CML was measured by ultra-high-performance liquid chromatography (UHPLC)–tandem mass spectrometry. Since this technique is highly demanding in terms of the quality and quantity of samples required for the experimental determination, we had to randomly split the 15 samples from each of the three tested groups into a subset of 10 on which to perform the UHPLC, while on the remaining 5 samples, experiments of Western blot, mtDNA content, and damage determination were carried out.

In brief, for UHPLC determination, 50 μL of sample (plasma or tissue homogenates) was hydrolysed with 500 μL of 0.6 M trichloroacetic acid and 50 mL of 6 M hydrochloric acid for 2 h at 60 °C. The analyses were performed on a UHPLC coupled to a triple quadrupole mass spectrometer (AB-Sciex Triple Quad 5500, Milan, Italy), equipped with a Turbo ion ESI source as reported in [24].

### 2.6. Western Blot Analysis

Western blot analyses were performed on frozen skeletal muscle (tibialis anterior) and heart (right ventricle) excised from the experimental animals. Briefly, around 40 mg of tissue was homogenized with a lysis buffer and centrifuged at 13,000× *g* for 25 min at 4 °C. Supernatants were then collected and total protein content was quantified using the Pierce™ BCA Protein Assay Kit (Thermo Fisher Scientific, Waltham, MA, USA). Then, 50 µg of proteins were loaded on a 10% or 4–12% sodium dodecyl sulphate–polyacrylamide gel for electrophoretic separation (SDS-PAGE) and transferred to a polyvinylidene difluoride membrane (GE10600023 Amersham™ Hybond^®^ P Western blotting membranes, PVDF or Merck KGaA, Darmstadt, Germany). The blots were then blocked with 10% non-fat dried milk for 1 h (A0830, PanReac Applichem, Darmstadt, Germany) and incubated with primary antibodies overnight at 4 °C (dilution 1:1000). 

The primary antibodies used were DRP1 (Abnova, Taipei City, Taiwan, #H00010059-M01), MFN2 (Cell Signaling Technology Inc., Danvers, MA, USA #9482S), PGC-1α (Novus Biologicals, Minneapolis, MN, USA #NBP1-04676PCP), TFAM (Cell Signaling Technology Inc., Danvers, MA, USA #7495S), SOD2 (MnSOD, Cell Signaling Technology Inc., Danvers, MA, USA #13194S), Peroxiredoxin 3, PRXIII (Ab Frontier, Seoul, Republic of Korea, #LFPA0030), β-actin (Sigma-Aldrich, St. Louis, MO, USA #A2066), NLRP3 (AdipoGene Life Sciences, San Diego, CA, USA, AG-20B-0014-C100), cleaved Caspase-1 (Cell Signaling Technology Inc., Danvers, MA, USA #89332), p47phox (nicotinamide adenine dinucleotide phosphate oxidase, NADPHox, Cell Signaling Technology Inc., Danvers, MA, USA #4301), and GAPDH (Cell Signaling Technology Inc., Danvers, MA, USA #2118). The next day, membranes were incubated for 1 h with a secondary antibody (Cell Signaling Technology Inc., Danvers, MA, USA anti-mouse #7076; anti-rabbit #7074) conjugated with HRP (dilution 1:10,000) and developed using the ECL detection system. Immunoreactive bands were analysed by the Bio-Rad Image Lab SoftwareTM 6.0.1 and results were normalized to standard diet set to 1. 

### 2.7. Determination of Relative mtDNA Copy Number

Total DNA was extracted from frozen skeletal muscle (tibialis anterior) and the heart (right ventricle). MtDNA relative copy number was determined by quantitative real-time polymerase chain reaction (qPCR), as reported in [25]. The primers 5′ AATCTACCATCCTCCGTGAAACC’3′ (nt 15,687–15,709) and 5′GCCCGGAGCGAGAAGAG3′ (nt 15,748–15,732) (GenBankTM accession number NC_005089.1) were used to obtain a 61 bp amplicon from the D-loop region of mtDNA; the primers 5′AGCCATGTACGTAGCCATCCA’3′ (nt 499–519) and 5′TCTCCGGAGTCCATCACAATG3′ (nt 579–559) (GenBankTM accession number NM_007393) were used to amplify a fragment of 80 pb of the nuclear β-actin gene. In total, 3 ng DNA was used as template.

### 2.8. Modified Purines Analysis

In order to evaluate the levels of oxidized purines in mtDNA of mice under investigation, total DNA was digested with formamidopyrimidine DNA glycosylase (Fpg) (New England Biolabs, Beverly, MA, USA), as reported in [26]. In total, 5 and 2.5 ng of Fpg-treated or untreated DNA was used as template to obtain a 590 bp-long amplicon encompassing the D-loop region of mtDNA. The primers were 5′GTGTTATCTGACATACACCATACAG3′ (nt 15,611–15,635) and 5′TGGGAACTACTAGAATTGATCAGGA3′ (nt 16,201–16,177) (GenBankTM accession number NC_005089.1). The levels of oxidized purines were expressed as the complement to 100% of the ratio between Fpg-treated and untreated band intensities. 

### 2.9. Reagents

If not otherwise reported, all reagents employed were from Sigma-Aldrich Company Ltd. (St. Louis, MO, USA).

### 2.10. Statistical Analysis

Data are shown as columns (mean ± SEM) for each experimental group. Statistical analysis was performed by one-way ANOVA, followed by Dunnett’s *post hoc* test. A *p*-value < 0.05 was considered significant. Statistical analysis was performed using GraphPad Prism^®^ software version 7.05 (San Diego, CA, USA).

## 3. Results

### 3.1. Characteristics of Dietary Interventions

Table 1 presents the properties as well as the energy supply (calories) of the diets given to the mice for 12 weeks. 

The HFHS diet had twice the number of calories compared to the HFr diet and the SD, which were isocaloric. The food intake, expressed in grams per day per mouse, was similar in all mice independently from the diet regimen. The HFr diet supplied a lower amount of fat than the HFHS diet, but a higher amount of sugar, fructose in particular, than the HFHS and SD (*p* < 0.05).

### 3.2. Effects of HFHS and HFr Diets on Metabolic Indicators

**Figure 1:**
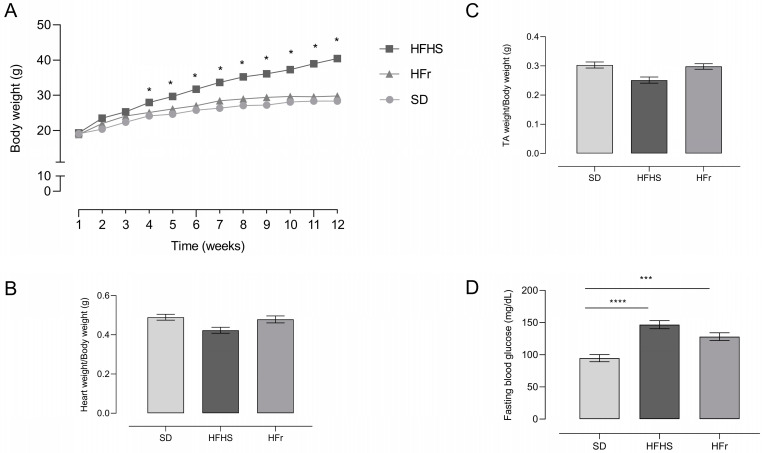
Body weight (**A**), heart weight (**B**), tibialis anterior (TA) skeletal muscle weight (**C**), and fasting blood glucose (**D**) in SD-, HFHS-, and HFr-fed mice at the end of the 12-week dietary protocol. Glucose level (mg/dL) in blood was analyzed after 12 h of fasting. Data are means ± SEM (*n* = 15 per group). The statistical significance level was set at *p* < 0.05. Statistical significance: * *p* < 0.05 vs. SD; *** *p* < 0.001 vs. SD; **** *p* < 0.0001 vs. SD.

Mice chronically fed with the HFHS diet for 12 weeks gained significantly more weight compared to HFr- and SD-fed mice (Figure 1A, *p* < 0.05 vs. SD). 

Heart weight (Figure 1B) and tibialis anterior skeletal muscle weight (Figure 1C), normalized to body weight, did not show any differences among the different groups.

After 12 weeks of dietary manipulation, the HFHS and HFr diets induced a significant increase in fasting blood glucose levels compared to SD-fed mice (Figure 1D). 

In order to have a general picture of glucose and insulin tolerance, an oral glucose tolerance test (OGTT) and insulin tolerance test (ITT) were performed. 

**Figure 2:**
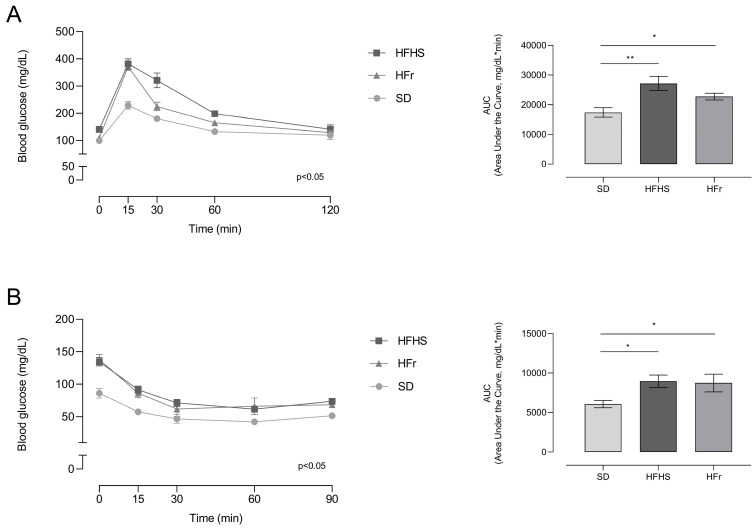
Effects of 12 weeks of dietary manipulation on oral glucose tolerance test (OGTT) and insulin tolerance test (ITT) responses. (**A**) OGTT was performed following a 12 h fasting period. Glucose was administered at a dose of 2 g/kg by gavage and blood glucose levels were detected by tail puncture after 15, 30, 60, and 120 min; (**B**) ITT was performed following a 6 h fasting period. After the measurement of basal glucose level, insulin was administered intraperitoneally at a dose of 0.75 IU of insulin/kg of body mass and blood glucose level was then evaluated by tail puncture after 15, 30, 60, and 90 min. Data are means ± SEM (*n* = 15 per group). Statistical significance: * *p* < 0.05 vs. SD; ** *p* < 0.01 vs. SD.

The HFHS and HFr diets determined an impaired response to both the OGTT and ITT, indicating a condition of glucose and insulin intolerance induced by the two diets (Figure 2). 

Lipid profiles and inflammatory and tissue damage parameters are reported in Table 2. Plasma levels of triglycerides and cholesterol significantly increased in HFHS- and HFr-diet-fed mice compared to SD-fed mice (*p* < 0.05). The HFr diet induced a further significant increase in cholesterol levels. IL-6 and LDH levels significantly increased in HFHS- and HFr-fed mice, suggesting, respectively, an increase in systemic inflammation and tissue damage (*p* < 0.05).

### 3.3. Inflammation and Oxidative Stress in Heart

**Figure 3:**
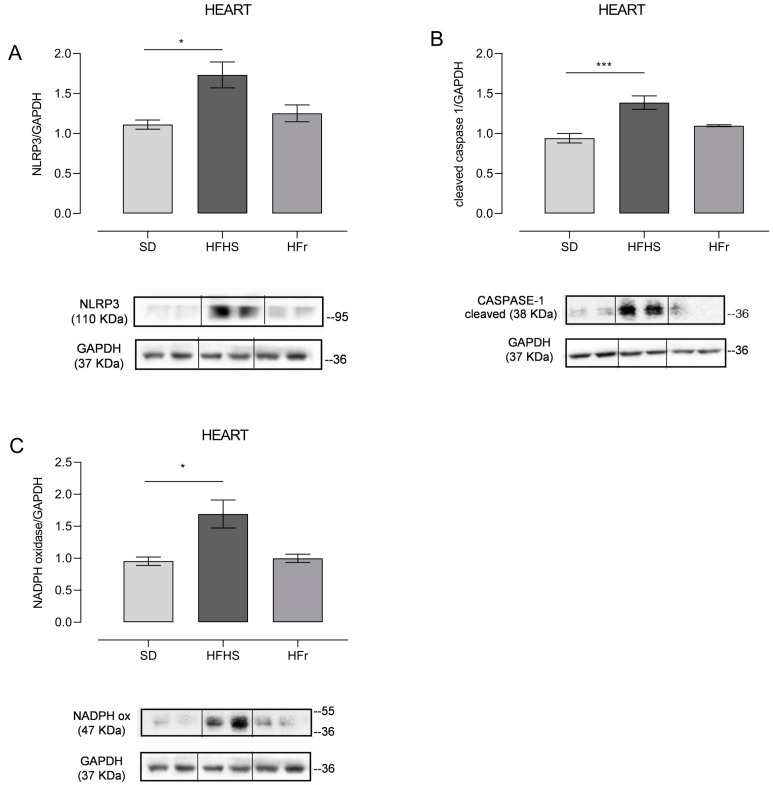
The effect of diets on inflammatory and oxidative stress markers in the heart. Western blot analyses were performed on frozen hearts, isolated at the end of the 12-week protocol. Inflammation was evaluated through measurement of NLRP3 (**A**) and cleaved Caspase-1 (**B**); oxidative stress was measured through determination of NADPH oxidase (**C**). (**A**) In the histogram, the ratio of the intensity of the NLRP3 vs. GAPDH bands is shown. A representative Western blot showing results for two mice from each group is shown in the inset. (**B**) The histogram shows the ratio between cleaved Caspase-1 vs. GAPDH intensities. As a representation, Western blot results obtained from two mice from each group are shown. (**C**) In the histogram, the ratio between the intensity of NADPH oxidase vs. the intensity of GAPDH bands is shown. A representative Western blot of two mice from each group is shown. (See also Supplementary Figures file S1). Data are means ± SEM (*n* = 5 per group). Statistical significance: * *p* < 0.05 vs. SD; *** *p* < 0.001 vs. SD.

Having demonstrated, through plasma measurements (Table 2), the presence of diet-induced inflammation, we decided to verify if this process was also specifically induced in the heart, using tissue from five mice randomly selected from each different diet group. The very small size of TA samples did not allow us to also perform these experimental determinations in TA tissue, but only in the heart samples. Expression level (Figure 3A) and activation of the downstream signaling of the NLRP3 inflammasome, in term of cleaved Caspase-1 (Figure 3B), were assessed by Western blot analysis in protein extracts from heart tissue.

Since a pro-inflammatory condition is usually accompanied by oxidative stress, we decided to also detect the presence of oxidative stress through evaluating the expression of NADPH oxidase, which catalyzes the production of a superoxide free radical by transferring one electron from the NADPH electron donor to the oxygen electron acceptor (Figure 3C).

NLRP3 expression significantly increased in HFHS-fed mice (*p* < 0.05), whereas the HFr diet did not induce significant changes compared to the SD (Figure 3A). Consequently, the expression of cleaved Caspase-1, namely the activated form of Caspase-1, responsible for the maturation of the pro-inflammatory cytokines IL-1β and IL-18, was, as expected, higher in the HFHS group (*p* < 0.001) (Figure 3B). Oxidative stress in the heart tissue of HFHS-diet-fed mice was demonstrated by a significant increase in the expression of NADPH oxidase compared to both the SD and HFr groups (*p* < 0.05) (Figure 3C). 

Since it has been shown that the onset of inflammation and oxidative stress may be induced by AGEs [16], we decided to verify the presence of these toxic compounds, which mostly originate from the diet through exogenous introduction or endogenous formation, in plasma, heart, and TA skeletal muscle samples.

### 3.4. AGEs in Plasma, Heart, and TA Muscle Tissues

We measured CML levels, as representative of AGEs, in homogenates from the plasma, heart, and TA skeletal muscle of 10 mice randomly selected from each diet group after the 12-week diet protocol.

**Figure 4:**
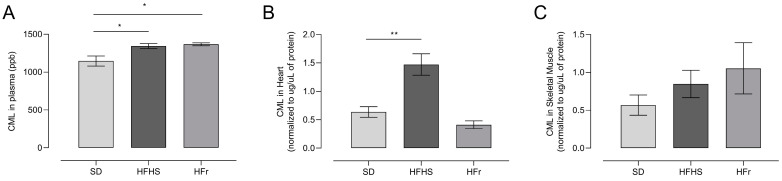
Advanced glycation end-products (AGEs) induced by the diets, analyzed through measurement of Nε-(carboxymethyl)-lysine (CML) at the end of the 12-week protocol. CML levels were evaluated by ultra-high-performance liquid chromatography (UHPLC)–tandem mass spectrometry in samples from plasma (**A**), heart (**B**) and TA skeletal muscle (**C**) homogenates from SD, HFHS, and HFr mice. A CML standard calibration curve was used, with concentrations of 10, 50, 100, 250, 300, and 500 μg L^−1^. Data are means ± SEM (*n* = 10 per group). Statistical significance: * *p* < 0.05 vs. SD; ** *p* < 0.01 vs. SD.

Plasma CML levels significantly increased (*p* < 0.05) in HFHS- and HFr-diet-fed mice compared to SD-fed mice (Figure 4A). CML levels were raised significantly in the hearts of HFHS-fed mice, but not in the hearts of HFr-fed mice (Figure 4B). CML levels did not show any significant difference in the TA muscle of either HFHS or HFr mice compared to SD-fed mice (Figure 4C).

Having verified HFHS-diet-related cellular oxidative stress in heart through the increase in NADPH oxidase expression as well in the presence of ROS-inducing AGEs, we decided to assess the potential presence of diet-related mitochondrial oxidative stress by analyzing the expression of mitochondrial antioxidant enzymes superoxide dismutase 2 (SOD2) and peroxiredoxin III (PRXIII).

### 3.5. Evaluation of Mitochondrial Oxidative Stress and Analysis of Relative mtDNA Content

**Figure 5:**
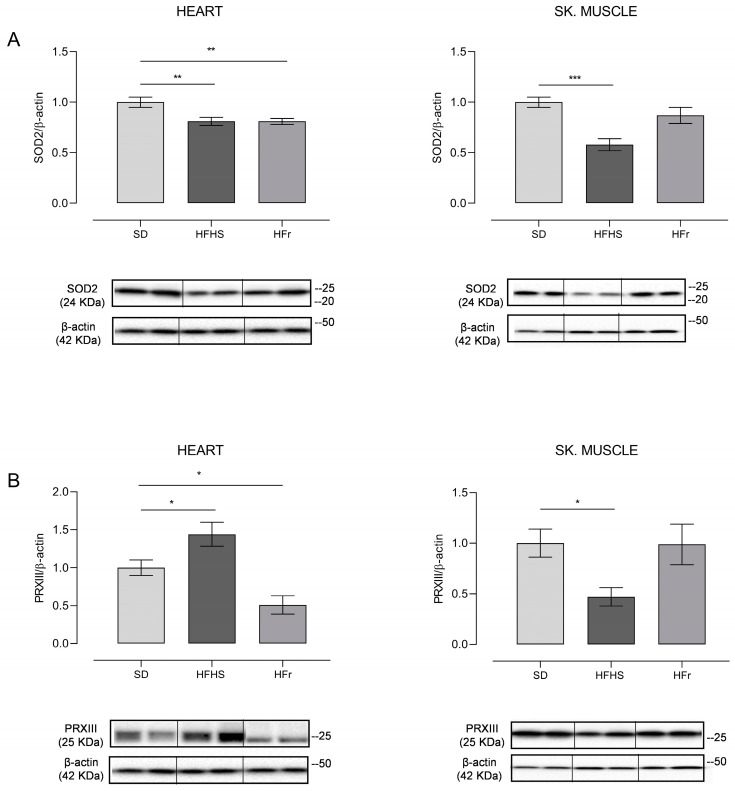
Western blot analysis of SOD2 (**A**) and PRXIII (**B**) in heart and TA skeletal muscle samples from SD, HFHS, and HFr mice. (**A**) In the histogram, the ratio of the intensity of the SOD2 vs. β-actin bands is shown. A representative Western blot showing the results for two mice from each group is shown in the inset. (**B**) The histogram shows the ratio between PRXIII vs. β-actin intensities. As a representation, Western blot results obtained from two mice from each group are shown. (**A**,**B**) Data shown are the results of Western blot experiments conducted at least in triplicate. Results were analyzed using a one-way ANOVA. Bars represent the mean values ± SEM (*n* = 5 per group). (See also Supplementary Figures files S1 and S2). Data are normalized against the value of the SD group, fixed as 1. Statistical significance: * *p* < 0.05 vs. SD; ** *p* < 0.01 vs. SD; *** *p* < 0.001 vs. SD.

Mitochondrial SOD2 is the antioxidant enzyme responsible for the neutralization of mitochondrial O_2_°^−^ or for protecting the mitochondria from externally produced ROS, while PRXIII is a mitochondrial ROS scavenger protein. Their expression was detected by Western blot experiments to evaluate the presence of mitochondrial oxidative stress in the heart and TA skeletal muscle from five mice randomly selected from each diet group at the end of the 12-week diet protocol. The results are presented in Figure 5. 

SOD2 showed a marked 20% significant decrease (*p* < 0.01) in HFHS and HFr heart samples in comparison to their SD counterparts, whereas a statistically significant 40% decrease compared to the SD samples (*p* < 0.001) was found in TA skeletal muscle samples of HFHS-fed mice only; a tendency to decrease was found in those from HFr-fed animals (Figure 5A). As for PRXIII expression, there was a significant 40% increase (*p* < 0.05) in the heart of HFHS animals with respect to the values found for the SD group, whereas it was significantly (50%) reduced (*p* < 0.05) in their counterparts from HFr mice. TA skeletal muscle samples presented a statistically significant 50% reduction (*p* < 0.05) in the HFHS group and no statistically significant change in the HFr group in comparison with SD animals in terms of PRXIII expression, suggesting a differential response to the nutritional regimens in the analyzed tissues (Figure 5B). 

Furthermore, the differential expression of the mitochondrial scavenger PRXIII in the heart and TA, depending on the adopted diet, prompted us to deepen the analysis at the mitochondrial oxidative stress level by dissecting mtDNA maintenance as well as mitochondrial biogenesis and dynamics. It has been demonstrated [26] that relative mtDNA content is deeply sensitive to oxidative stress induced by different causes inside the organelles.

**Figure 6:**
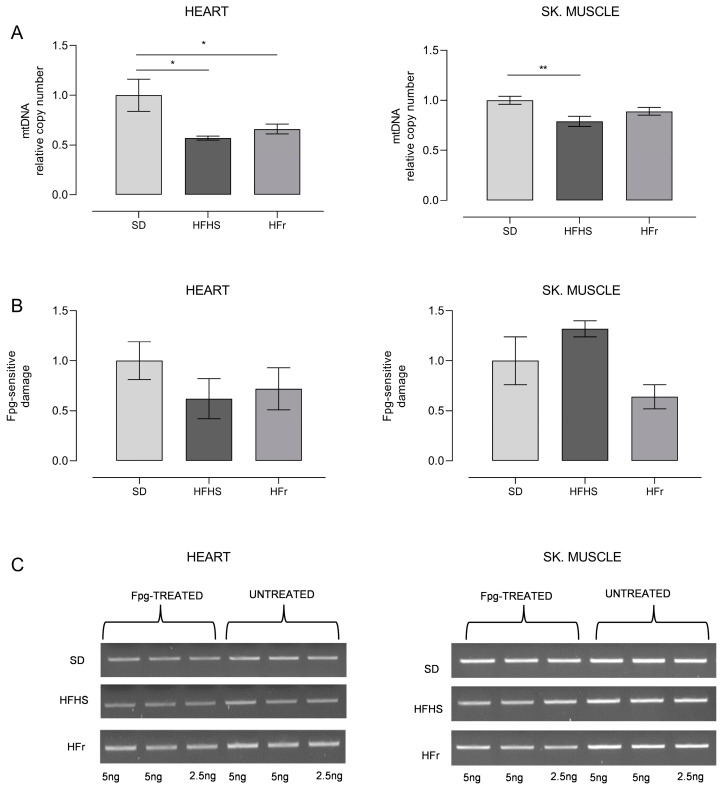
Relative mtDNA content and oxidative damage to mtDNA. (**A**) Relative mtDNA content in heart and TA skeletal muscle samples from SD, HFHS, and HFr mice. Data are normalized vs. values for SD mice, fixed as 1. (**B**) Incidence of oxidatively modified purines at the D-loop in samples of heart and TA skeletal muscle. Bars represent the ratio between the intensities of treated and untreated bands. (**C**) Representative agarose gel showing amplicons obtained from Fpg-treated and untreated total DNA. (See also Supplementary Figures files S1 and S2). Data are means ± SEM (*n* = 5 per group) and are normalized against the values for SD group, fixed as 1. Statistical significance: * *p* < 0.05 vs. SD; ** *p* < 0.01 vs. SD.

Therefore, we determined by qRT-PCR the relative content of mtDNA in both heart and TA skeletal muscle samples from the three groups of mice; the results are reported in Figure 6A. 

We also detected the frequency of oxidatively modified bases, mainly 8-hydroxydeoxyguanosine (OH8dG), in a specific region of mtDNA, namely the control region, spanning over the D-loop of mtDNA and crucial for the replication and transcription of mtDNA [27]. The results of this analysis, carried out through digestion with the OH8dG-sensitive enzyme Fpg of the amplified region, are shown in Figure 6B,C. 

As for the heart samples, both HFHS and HFr diets implied, respectively, a significant loss of mtDNA content, of 42% and 34%, in comparison with SD samples (*p* < 0.05). Conversely, only the HFHS diet induced a statistically significant (21%) loss of mtDNA in TA skeletal muscle (*p* < 0.01), while the HFr diet did not imply any significant change (Figure 6A). The detection of the incidence of OH8dG in the D-loop region of mtDNA showed the presence of a n.s. 40–30%-decreased level of oxidized base in the heart in, respectively, both HFHS- and HFr-fed mice. In TA skeletal muscle samples, unexpectedly, there was a n.s. 32% increase in the frequency of oxidized bases in HFHS-fed mice only, whereas in their counterparts from HFr-fed mice, there was a n.s. 36% decrease in the oxidative damage assayed (Figure 6B,C).

### 3.6. Evaluation of Mitochondrial Biogenesis and Dynamics

**Figure 7:**
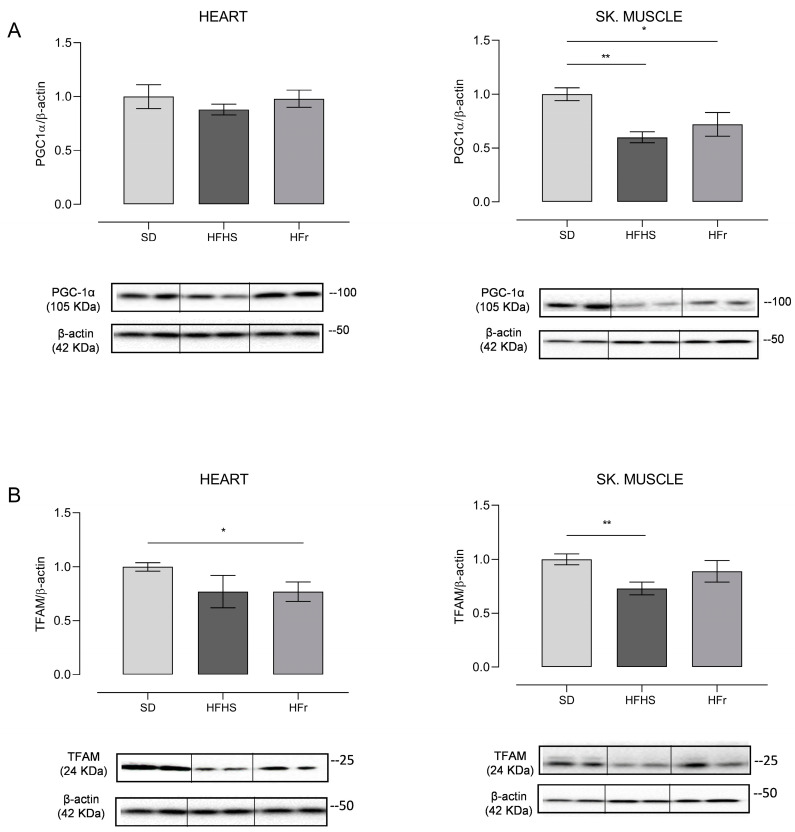
Western blot analysis of proteins involved in mitochondrial biogenesis PGC-1α (**A**) and TFAM (**B**) in heart and TA skeletal muscle samples from SD, HFHS, and HFr mice. (**A**) In the histogram, the ratio of the intensity of the PGC-1α vs. β-actin bands is shown. A representative Western blot showing the results for two mice from each group is shown in the inset. (**B**) The histogram shows the ratio between TFAM vs. β-actin intensities. As a representation, Western blot results obtained from two mice from each group are shown. (**A**,**B**) Data shown are the results of Western blot experiments conducted at least in triplicate. Results were analyzed using one-way ANOVA. Bars represent the mean values ± SEM (*n* = 5 per group). (See also Supplementary Figures files S1 and S2). Data are normalized vs. the values for the SD group, fixed as 1. Statistical significance: * *p* < 0.05 vs. SD; ** *p* < 0.01 vs. SD.

Having verified the different effects of the diets on relative mtDNA content according to the tested organ, that is on the maintenance of the mitochondrial genome, we decided to deepen the study at the mitochondrial biogenesis level, by analysing the expression of peroxisome proliferator-activated receptor gamma coactivator 1-alpha (PGC-1α) and mitochondrial transcription factor A (TFAM) proteins, which are key factors in the process, through Western blot experiments. The obtained results are reported in Figure 7. 

The amount of PGC-1α, which is the master regulator of mitochondrial biogenesis, did not show any significant change in heart samples from both the HFHS and HFr diets. On the contrary, the expression of PGC-1α presented a statistically significant decrease in TA skeletal muscle of, respectively, 40% in the HFHS diet (*p* < 0.01) and of 30% in the HFr counterpart (*p* < 0.05) (Figure 7A). As for the mitochondrial histone-like protein, that is TFAM, in the heart, its amount presented a n.s. decrease of 23% in HFHS samples and a significant decrease of 27% in HFr samples (*p* < 0.05). In the TA skeletal muscle, conversely, only in samples from HFHS-fed mice was there a statistically significant (27%) decrease in the amount of TFAM (*p* < 0.01), while in samples from HFr-fed mice, there was no statistically significant change (Figure 7B).

**Figure 8:**
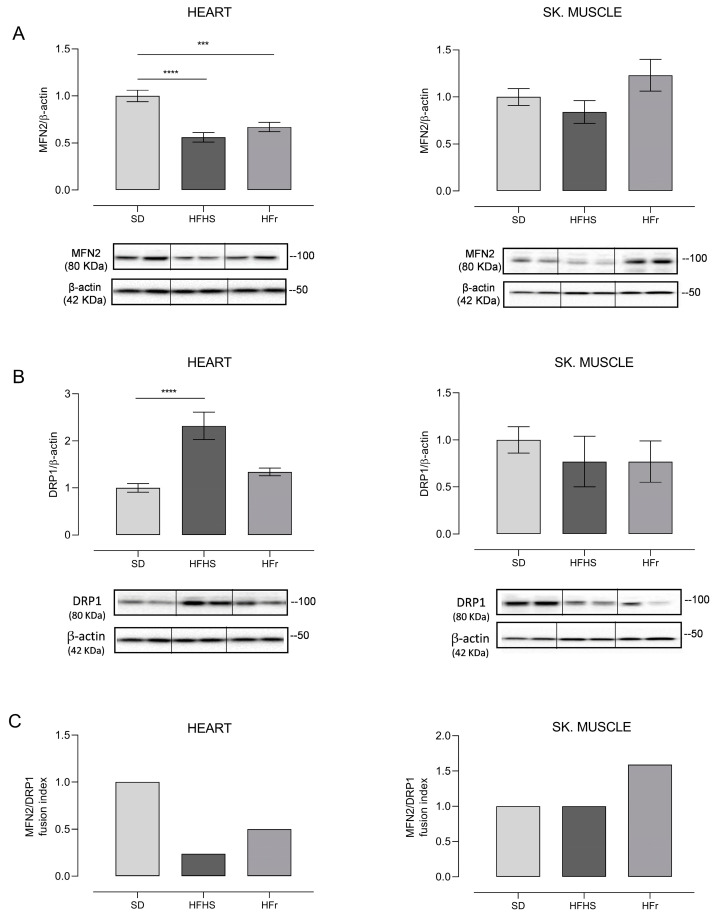
Western blot analysis of proteins involved in mitochondrial dynamics. Amounts of MFN2 and DRP1 proteins and fusion index (FI) in heart and TA skeletal muscle samples from SD, HFHS, and HFr mice. (**A**) In the histogram, the ratio of the intensity of the MFN2 vs. β-actin bands is shown. A representative Western blot showing the results for two mice from each group is shown in the inset. (**B**) The histogram shows the ratio between DRP1 vs. β-actin intensities. As a representation, Western blot results obtained from two mice from each group are shown. (**A**,**B**) Data shown are the results of Western blot experiments conducted at least in triplicate. Results were analyzed using one-way ANOVA. Bars represent the mean values ± SEM (*n* = 5 per group). (See also Supplementary Figures files S1 and S2). Data are normalized against the value of the SD group, fixed as 1. Statistical significance: *** *p* < 0.001 vs. SD; **** *p* < 0.0001 vs. SD. (**C**) Fusion index (FI). Bars represent the mean value of the fusion index (FI) value as the ratio between MFN2 and DRP1 for each of the examined mice. Data are normalized against the values for the SD group, fixed as 1.

Another fundamental mitochondrial process which needed to be assessed in order to verify the effects of the diets on the organelles was the balance of mitochondrial dynamics, analysed through the determination of the expression of mitofusin 2 (MFN2) and dynamin-related protein 1 (DRP1) by Western blot experiments and through the calculation of their respective fusion index (FI), i.e., the ratio between MFN2 and DRP1 values. The results of these experimental approaches are reported in Figure 8. 

The expression of MFN2, indicative of fusion activity, determined in heart samples notably showed a significant decrease, respectively, of 44% in the HFHS diet (*p* < 0.0001) and of 33% in the HFr diet (*p* < 0.001) with respect to the SD. Responses to the diets in the TA skeletal muscle, conversely, were very different, as HFHS resulted in a tendency to decrease fusion, although with no significant change, whereas HFr elicited a tendency to increase MFN2, suggestive of a very active fusion process (Figure 8A). The activity of fission was evaluated through the determination of DRP1 expression; in heart samples, a remarkable, significant 132% increase induced by the HFHS diet (*p* < 0.0001) versus a n.s. tendency to increase evoked by its HFr counterpart were found, in comparison with SD. In the TA skeletal muscle, there were no significant changes, although in HFHS diet samples, there was a tendency to decrease fission, which was further enhanced in HFr samples (Figure 8B). The calculation of the FI identified very different effects of the diets on the balance of mitochondrial dynamics, shedding light on clear tissue-specificity. In fact, the FI markedly decreased in the hearts of mice treated with both diets in comparison with SD, but the final figures were derived from their different effects on fission, since fusion was similarly significantly reduced by both HFHS and HFr, while fission showed a specific dramatic increase induced by HFHS alone. In TA skeletal muscle samples, the diet-induced change in FI with respect to their SD counterparts was even more pronounced because HFHS did not evoke any change in the final value of the marker, while HFr led to an increased value, mainly due to a notable n.s. increase in fusion (Figure 8C).

## 4. Discussion

Having verified the very complicated relationships among the assayed markers, depending on the kind of diet administered and on the examined tissue, we decided to discuss the effects of the diets, splitting the results by the tested organs to highlight the tissue-specific differential responses.

### 4.1. Systemic Profile in HFHS Diet

The HFHS diet, because of its doubled amount of calorie intake in comparison to the SD and HFr regimens, induced a marked, progressive, and statistically significant increase in body weight, fully demonstrating its obesogenic capacity, although without any change in heart and TA weights. Our results are consistent with those from another study reporting the effect on body weight of three different diets, namely HFHS, HF, and HS, administered to rats for 20 weeks [28]. In HFHS-fed mice, diet-related obesity was accompanied, at the end of the 12-week protocol, by an increased fasting blood glucose level and increased OGTT values with respect to the values of their SD-fed counterparts, indicative of the induced hyperglycemia. Furthermore, the HFHS diet also led to marked insulin resistance, as shown by the results of the ITT, which are consistent with literature reports [29] indicating HFHS as the most effective diet for the induction of insulin resistance [30].

The lipid profile of the HFHS-fed mice described here (Table 2) clearly showed their increased intake of diet-derived triglycerides and cholesterol.

As for the total cholesterol concentration in the plasma, the raised value reported here agrees with data in the literature, showing an increase in total cholesterol concentration in different HFHS-fed mice strains [31]. Furthermore, a large consistency between our metabolic results and those of similar studies is evident since HF feeding of adult rats was shown to significantly increase body weight and total adipose tissue weight with decreased skeletal muscle mass, confirming the condition of obesity and insulin resistance induced by a HF diet [32]. A feature of obesity is also chronic low-grade inflammation of peripheral tissues [4]. We decided to assess whether HFHS diet-induced obesity presented such a condition. The increased plasma concentration of IL-6 (Table 2) in HFHS-fed mice verified the appearance of systemic inflammation in the rodents and was consistent with literature reports showing the presence of increased systemic inflammation in HF-fed mice only from the 12th week of diet onwards [31,33]. Furthermore, the raised plasma concentration of LDH in the HFHS-fed mice studied here demonstrated that the inflammatory response also implied increased tissue damage. Worthy of special mention is a recent study that originally used multivariate data analyses to compare metabolic indicators in plasma and other tissues from rats treated with one out of three different diets, highlighting that the metabolic changes induced largely depended on the diet composition, that is on the different proportions of cholesterol, fat, and sugar [34]. The next step was the evaluation of the diet-induced activation of the NLRP3 inflammasome, which was carried out only in the heart because the very small size of the TA samples did not allow us to also perform these experimental determinations in TA tissues. For this reason, the following section will discuss all the experimental data deriving from the hearts of HFHS-fed mice. 

#### 4.1.1. Hearts of HFHS Mice

Expression of the NLRP3 protein and the activation of its downstream signaling, leading to activated Caspase-1, were shown to be increased in HFHS-fed hearts, confirming the diet-induced activation of NLRP3 reported in the kidneys and livers of mice treated with a HFHS diet for 16 weeks [35]. Due to the relevance of ROS for the activation of the NLRP3 inflammasome [36], we decided to assess the expression of NADPH oxidase, which is an important source of ROS, in HFHS-fed hearts, and found a diet-increased amount of the protein, confirming a similar report in 12-week HFHS-fed rats [22]. Since it has been reported that the activation of NADPH oxidase can be obtained by AGEs [16], the next step was monitoring the presence of a prevalent AGE, namely CML, in different tissues from HFHS-fed mice. Indeed, the diet induced a statistically significant increase in CML levels in the plasma and the heart, but not in skeletal muscle, and raised CML could have induced the NADPH oxidase expression found in the heart. The accumulation of CML in the heart, demonstrated here by UHPLC–tandem mass spectrometry, is another original and remarkable finding of this study, since the previous, consistent report about this issue only detected the degree of AGE-modified proteins by competitive enzyme-linked immunosorbent assay in heart cytoplasmic extracts [37]. It is firmly established that AGEs can induce the production of ROS, also affecting the functionality of the mitochondrial respiratory chain [16]; therefore, we evaluated the presence of mitochondrial oxidative stress by the determination of the expression of two major mitochondrial antioxidant enzymes, that is SOD2 and PRXIII. The expression of SOD2 showed a marked 20% significant decrease in HFHS heart samples, whereas PRXIII expression presented a significant 40% increase in the same samples. The latter result is particularly remarkable because it is exclusively heart-specific, and it will later be discussed together with other heart-related findings that consistently support our novel hypothesis. The unexpected HFHS-reduced expression of SOD2 will be explained further on through the analysis of the diet’s effects on mitochondrial biogenesis. Overall, these latter results indicate that diet-induced oxidative stress was very efficaciously counteracted in the heart mitochondria, since there was no evidence of the typical changes that appear alongside a mitochondrial oxidative stress response. This is consistent with a report demonstrating a decreased production of mitochondrial H_2_O_2_ in the hearts of HFHS-fed rats [21]. However, we decided to assess mtDNA maintenance and damage, as we previously demonstrated that changes in relative mtDNA content constituted a reliable and sensitive marker of oxidative stress effects on the mitochondria [25,38]. We found a significant 42% loss of mtDNA content and the presence of a n.s. 40% decreased level of oxidized base OH8dG in HFHS-fed heart samples in comparison with their SD counterparts. This means that in HFHS hearts, mtDNA, although decreased in terms of content, presented high-quality molecules, even less damaged than those in SD hearts. The present data are the first to report the effects of a HFHS diet on mtDNA content and damage level in the heart; a previous study analysed the effects of a HF diet on the gastrocnemius skeletal muscle and liver in terms of these parameters, presenting results consistent with ours. This same report also detected an effect of a HF diet on the amount of the master regulator of mitochondrial biogenesis that is PGC-1α and of the histone-like mtDNA protein that is TFAM, describing a consistent diet-induced reduction in their amounts as well as in those of SOD2 [39]. Our findings in HFHS hearts showed an unchanged content of PGC-1α and a n.s. 23% decrease in the amount of TFAM, which together with the above-reported 20% decrease in the amount of SOD2 strongly suggests that diet-induced oxidative stress affected mitochondrial biogenesis in the heart quite severely. The present results further confirm that SOD2 expression was more controlled by mitochondrial biogenesis regulation than by the oxidative stress response [27]. The last major mitochondrial function examined in our study was dynamics, involved, together with mitochondrial biogenesis and mitophagy, in the complex and fundamental network of mitochondrial quality control (MQC) processes. The fine-tuned balance between fission and fusion allows mitochondrial dynamics to constantly adapt the morphology and activity of the mitochondria to the always changing bioenergetic and biosynthetic demands of cells. Mitochondrial fission is mainly regulated by DRP1, while mitochondrial fusion is regulated by MFN2 [40]. In HFHS hearts, we found a significant 44% decrease in the expression of MFN2 with respect to the SD and a remarkable significant 132% increase in DRP1 expression in comparison with the SD, leading to an FI equivalent to 25% of its SD counterpart. These results clearly indicate that fusion was markedly reduced, and fission was dramatically activated by the diet, with the latter facilitating the elimination of oxidatively damaged mtDNA molecules so that the remaining ones, although reduced in terms of copy number, were of the best available quality. This means that the heart was affected by diet-induced heavy oxidative stress (increased presence of AGEs) in a tissue-specific manner, leading to a strong expression of NADPH oxidase, generating extra-mitochondrial ROS. Diet-induced heavy oxidative stress also resulted in reduced mitochondrial biogenesis, sensitive to ROS produced by the increased utilization of diet-derived triglycerides through β-oxidation. However, the heart efficiently counteracted these diet-related alterations through a new balance of mitochondrial dynamics and an increased expression of PRXIII. The latter finding is consistent with the novel hypothesis according to which PRXIII cooperates with MQC processes in the heart through the regulation of mitochondrial ROS removal and oxidative damage prevention as well as clearance of damaged mitochondria by mitophagy modulation [41]. These different actions resulted in the heart’s comprehensive intrinsic ability to adapt to the oxidative stress imposed by the HFHS diet. Further support to this hypothesis comes from a previous study demonstrating the heart’s adaptation to oxidative stress induced by the nutrient overload of a HFHS diet through the upregulation of mitochondrial thioredoxin reductase-2 and improved preservation of the mitochondrial redox state [21]. It needs also to be highlighted that mitochondrial fission, which requires DRP1, is essential for the segregation of damaged mitochondria in a DRP1-dependent mitophagy mechanism that has been deeply scrutinized in the heart [42] and that might very consistently explain the present diet-induced increase in the amount of DRP1 and the sequential effects on the quantity and quality of mtDNA. A notable confirmation of the influence exerted by the adopted nutritional regimen on heart mitochondria derives from the very recent study demonstrating how the energetic state of heart, through regulating the assembling or stability of mitochondrial supercomplexes, affected the abundance of these functional aggregations [43]. From a functional viewpoint, a fairly recent study demonstrated that only one month on the HFHS diet affected the energetics in the beating heart and maximal ATP production in isolated mitochondria, much before the 4 months of HFHS diet after which cardiac structural remodeling (i.e., myocyte hypertrophy, myocardial hypertrophy, interstitial fibrosis) had been reported [44]. Our results about an overall compensation for HFHS-induced heavy oxidative stress occurring in heart mitochondria after a 12-week diet suggest that it might delay the negative effects on the heart’s structure, so that structural remodeling appears only after 4 months (16 weeks) of a HFHS diet but will eventually lead to metabolic heart disease.

#### 4.1.2. TA of HFHS Mice

The discussion of the results of HFHS-fed TA samples begins with the determination of CML levels, as representative of AGEs, which were not statistically changed by the diet, showing a very different response in comparison to the heart. Also, this was a novel finding of the present study and might open new lines of research about the tissue-specific localization of AGEs and their effects. Subsequent research on mitochondrial oxidative stress markers, namely the amounts of SOD2 and PRXIII, showed a 40% decreased value for SOD2 and a 50% decreased value for PRXIII. Again, both values were very different from their heart counterparts and suggested a TA-specific response. The assessment of the relative content and oxidative damage of mtDNA demonstrated a 21% decreased mtDNA content, less severe than the 42% reduction in its content in the heart, whereas there was a n.s. 30% increased level of OH8dG in comparison with its SD counterpart. The present results are consistent with those of a study in mice that also reported a decreased mtDNA content and an increased mtDNA damage in HFD-fed mixed gastrocnemii [39]. Therefore, in comparison to the heart, TA mtDNA quantity decreased to a lesser extent, but its quality was worse, which suggests a more significant or less efficiently counteracted mitochondrial oxidative stress in the skeletal muscle, also supported by the increased generation of mitochondrial H_2_O_2_ in red gastrocnemii from HFHS-fed rats [21]. In TA, diet-induced mitochondrial oxidative stress affected mitochondrial biogenesis more heavily than it did in the heart, as demonstrated by the 40% and 30% decreases, respectively, in the amounts of PGC-1α and TFAM. Notably, only the HFHS diet induced a tendency to decrease in both fusion (MFN2) and fission (DRP1) and the respective FI. Therefore, in the TA, HFHS-induced heavy oxidative stress diminished the mitochondrial biogenesis and MQC processes, as indicated by the 50% decreased PRXIII amount, and led to mtDNA damage more heavily than it did in the heart. These data are supported the absence of TA adaptation to HFHS and this conclusion is reinforced by a consistent analysis carried out in a previous paper about the HFHS diet’s effects on the heart and red gastrocnemius which highlighted the skeletal muscle’s specific inability to augment its endogenous antioxidant network [21]. It also needs to be pointed out that, because of the insulin resistance associated with HFHS-induced obesity, both the uptake and utilization of glucose in the skeletal muscle are significantly reduced, shifting to fatty acid (FA) oxidation the production of ATP for most metabolic processes and for contraction. Such a condition is also referred to as metabolic inflexibility or a disrupted “glucose-fatty acid cycle” and leads, through prominent FA oxidation, to the accumulation of toxic products of fat metabolism and pro-inflammatory cytokines, which further exacerbate insulin resistance. Finally, a HFHS diet contributes to skeletal muscle atrophy characterized by a decrease in myofibrillar proteins and loss of muscle mass and strength [45], which very efficiently mimic one of the most devastating age-related pathologies. Altogether, these data reinforce the conclusion that certainly the TA, and likely skeletal muscle in general, is characterized by a reduced ability to adapt to changes in endogenous or environmental conditions exemplified by the HFHS diet, which is particularly striking in comparison with the remarkable adaptability of the heart. The reduced adaptability of the tibialis to HFHS diet-induced heavy oxidative stress is also supported by a paper reporting a decrease in ATP synthesis in the tibialis skeletal muscle exposed to only 6 weeks of a HF diet, suggestive of a mild OXPHOS uncoupling [46]. 

### 4.2. HFr Diet

Several recent studies have pointed out a causal relationship between excessive fructose intake and metabolic diseases, including NAFLD, CVD, and T2D. In particular, fructose is a more potent inducer of hepatic de novo lipogenesis (DNL) than glucose through the conversion of excess carbons into lipids [47], thus leading to overweight and obesity. This dangerous outcome also makes the HFr diet an important object of research for its metabolic and pathological consequences. 

The HFr diet administered in our study was isocaloric with the SD, which explains the absence of weight increase both at the whole body as well as at the heart and TA levels. However, the considerable 70% intake of total calories from carbohydrates, of which 60% were from fructose, accounts for the significant increase in fasting blood glucose level and the induced hyperglycemia and marked insulin resistance shown, respectively, by the OGTT and ITT, after 12 weeks of the protocol, with respect to SD-fed mice. Our results are consistent with literature reports showing that excessive fructose increased indices of insulin resistance, visceral adiposity, DNL, and atherogenic dyslipidemia. In particular, fructose may facilitate DNL through multiple mechanisms leading to liver morbidity, which has been clearly verified in recent years [48]. The lipid profile of the HFr-fed mice described here (Table 2) presented significantly increased plasma levels of triglycerides and cholesterol in comparison with their SD-fed counterparts. Worthy of attention is the more than threefold rise in total cholesterol value seen in the HFr diet, significant in comparison to both its SD and HFHS counterparts. It clearly demonstrates, together with the increased triglycerides level, the strong induction of lipogenesis due to excessive fructose intake, which was also reported by a recent study conducted by some of us on mice fed with a fructose-rich diet [49]. The significant increases in plasma concentrations of IL-6 and LDH (Table 2) in HFr-fed mice demonstrated that this diet also induced both systemic inflammation, although less severe than that of the HFHS-fed counterparts, and notable tissue damage, confirmed by similar results for IL-6 and other plasma inflammatory markers [45]. 

The next step was the evaluation of the diet-induced activation of the NLRP3 inflammasome, which was carried out only in the heart because the very small size of the TA samples did not allow us to also perform these experimental determinations in TA tissue. Therefore, the following section will discuss all the experimental data deriving from the hearts of HFr-fed mice.

#### 4.2.1. Hearts of HFr Mice

A peculiar difference between the HFHS and the HFr diets was that the latter did not induce an increase in the expression level and activation of the downstream signaling of the NLRP3 inflammasome since the amounts of both NLRP3 and cleaved Caspase-1 did not show significant changes with respect to their SD counterparts. Since the activation of the NLRP3 inflammasome also requires the involvement of ROS [30], we decided to analyze the expression of an important source of ROS, that is NADPH oxidase, in HFr-fed hearts, and found an unchanged amount of the protein, consistent with the absence of activated NLRP3. However, an above-quoted study describing the effects of a fructose-rich diet in the murine heart demonstrated the increased presence of oxidative stress markers [49] and drove us to assess the level of CML, chosen as the representative of AGEs, which can also induce oxidative stress by affecting the functionality of the mitochondrial respiratory chain [16]. The HFr diet induced a statistically significant increase in CML levels only in the plasma, but not in the heart or the TA, as if the latter two tissues had been able to counteract the noxious effects of the sugar up to the end of our treatment, namely 12 weeks, but would likely have lost this ability after a longer exposition to a fructose-rich diet, like the 24 weeks in the above-mentioned study [49]. Nevertheless, the present quantitative determination of CML in the heart by UHPLC–tandem mass spectrometry is another original and significant finding of this study. In order to find out whether the HFr diet affected mitochondrial functionality leading to mitochondrial oxidative stress, we determined the expression of SOD2 and PRXIII. SOD2 showed a marked, significant 20% decrease in HFr heart samples and an even more remarkable 50% significant decrease was found for PRXIII expression in comparison to its SD counterparts. The HFr-induced decrease in SOD2 expression was comparable with that induced by the HFHS diet and it might have been due, similarly to what was discussed in the corresponding section, to a decrease in mitochondrial biogenesis, also controlling SOD2 expression. As for the very marked decrease in PRXIII expression in the HFr heart, this was very different from the significant 40% increase shown by the protein in HFHS-fed animals, explained in the corresponding section [41]. The strikingly reduced expression of PRXIII in the HFr heart suggests that the molecular trigger, activated in the HFHS diet and likely related to diet-induced heavy oxidative stress, was not elicited in the HFr diet, further demonstrating the heart’s sensitivity to the adopted nutritional regimen. However, it was necessary to assess the potential presence of mitochondrial oxidative stress through the determination of the relative mtDNA content and the frequency of oxidized base OH8dG. In HFr-fed heart samples, we found a significant 30% loss of mtDNA content and the presence of a n.s. 28% decreased level of OH8dG in comparison with their SD counterparts. This means that, similarly to what we found in HFHS hearts, mtDNA, although decreased in terms of content, presented high-quality molecules, less damaged than even those found in SD hearts. These are the first data about HFr diet’s effects on mtDNA content and damage level in the heart and they prompted us to also analyze the influence of the diet on the amounts of PGC-1α and TFAM. Similarly to what is described here for the HFHS diet, PGC-1α showed an unchanged content and TFAM showed a significant 27% decrease in its amount, which, together with the above-reported 20% decreased SOD2 amount, strongly suggests that the HFr diet affected mitochondrial biogenesis in the heart quite severely. We then analyzed the influence of the HFr diet on mitochondrial dynamics, finding a significant 33% decrease in the expression of MFN2 with respect to the SD and a n.s. tendency for DRP1 expression increase in comparison with the SD, leading to an FI equivalent to 50% of its SD counterpart. Therefore, fusion was markedly reduced, and fission was quite strongly increased by the diet, implying that, although at a lower degree than in the HFHS heart, fission was active in facilitating the elimination of oxidatively damaged mtDNA molecules so that the high quality of the remaining ones could allow for their unaltered performance. This means that a 12-week HFr diet affected the heart in a tissue-specific manner, eliciting marked mitochondrial oxidative stress, although not affecting the organelle’s functionality severely. In fact, the induced alterations were quite efficaciously counteracted by a tissue-specific response involving a marked tendency for mitochondrial fission increase, facilitating the physical elimination of oxidized mtDNA molecules. These results further support the heart’s intrinsic ability to adapt to changing conditions, already seen with the oxidative stress imposed by HFHS diet.

#### 4.2.2. TA of HFr Mice

The discussion of the results of HFr-fed TA samples begins with the determination of CML levels, as representative of AGEs, which were not statistically changed by the diet, as was also found in the heart. This was a novel finding of the present study, which demonstrated that the response to the HFr diet, in terms of the accumulation of AGEs, was the same in both assayed tissues, in contrast with what was seen for the HFHS diet. As for the HFr-induced changes in the expression of mitochondrial stress markers in the TA, the amount of SOD2 remained unchanged, as did that of PRXIII, in comparison to their SD counterparts, suggestive of very mild mitochondrial oxidative stress and not eliciting any special compensatory response. Both TA values were very different from their heart counterparts and suggested a TA-specific response. The assessment of the relative content and oxidative damage of mtDNA showed an unchanged mtDNA content and a n.s. 36% decreased level of OH8dG in comparison with their SD counterparts. The conclusion that can be drawn for the TA is clearly different from that for heart, as the quantity of mtDNA did not change and its quality was very high as the TA adapted more efficiently than the heart to HFr-induced stress. The latter indication was further supported by the significant 28% reduction in, and unchanged amounts of, respectively, PGC-1α and TFAM in the TA, which were differently affected by the HFr diet with respect to their heart counterparts, suggesting a very mild effect on mitochondrial biogenesis and confirming the strong quantitative relationship between mtDNA copy number and TFAM amount [27,50]. The analysis of the effects of the HFr diet on mitochondrial fission (DRP1), fusion (MFN2), and the respective FI demonstrated that there were no significant changes in these parameters in the TA, although there was a novel tendency for increase in fusion, resulting in an increase in FI that had not previously been found, which could be explained considering that MFN2 and other proteins involved in sarcoplasmic reticulum–mitochondria interactions were all overexpressed in prediabetic WT mice, namely fructose-rich-diet-treated mice [51]. Altogether, these data support the conclusion that the HFr diet had a less severe effect on the TA than the HFHS diet, which overall elicited mild counteractions, exemplified by a tendency for increased fusion, thus showing the adaptability of both the heart and TA to this diet. Recent metabolomic analysis studies support the present data on differential tissue-specific sensibilities to HFHS or HFr diets, suggesting a crosstalk between the analyzed organs by means of specific metabolites [52].

Further work will allow for a dissection of the detailed mechanisms leading to the different tissue-specific responses elicited by each of the assayed diets in heart and TA. These studies will be also relevant in view of the possible therapeutic interventions aiming to prevent or delay the onset/development of diet-induced cardiometabolic disorders and diet-related skeletal muscle atrophy.

## 5. Conclusions 

In summary, our data provide novel evidence that, aside from the extensively described effects of HFHS and HFr diets on glucose and insulin homeostasis, these diets differentially and significantly affect the metabolism of the heart and skeletal muscle. Tissue-specific differential responses to each assayed diet strictly depended on the specific relevance of diet-induced oxidative stress. The present results allowed us to detect the remarkable adaptability of the heart to HFHS-induced heavy oxidative stress, with important effects on the presence of AGEs and influence on mitochondrial biogenesis, efficaciously counteracted by changes in mitochondrial dynamics and MQC activities. On the other hand, we pointed out the absence of adaptation to the HFHS diet in the TA, revealed by a heavy reduction in mitochondrial biogenesis that was not efficaciously counteracted by mitochondrial changes. Conversely, HFr-induced mild oxidative stress elicited tissue-specific effects that were counteracted by different changes in both tissues. Specifically, in the heart, quite a severe decrease in mitochondrial biogenesis was counteracted by a tendency for increased fission, whereas in the TA, a mild reduction in mitochondrial biogenesis was counteracted by a tendency for increased fusion, thus showing the differential adaptability of both the heart and TA to the latter diet.

## Figures and Tables

**Table 1:** Food intake and calorie intake of mice fed SD, HFHS, or HFr diet for 12 weeks.

	SD (*n* = 15)	HFHS (*n* = 15)	HFr (*n* = 15)
Diet energy supply (kcal/g)	3.850	5.560	3.850
Food intake (g/day/mouse)	2.889 ± 0.308	3.104 ± 0.553	3.109 ± 0.411
Total calorie intake (kcal/day/mouse)	11.124 ± 1.186	17.259 ± 3.072 *a*	11.968 ± 1.583 *b*
Fat calorie intake (kcal/day/mouse)	0.478 ± 0.051	10.010 ± 1.782 *a*	0.515 ± 0.068 *b*
Sucrose or fructose calorie intake (kcal/day/mouse)	0.000 ± 0.000	1.291 ± 0.230 *a*	7.181 ± 0.950 *a*,*b*

Data are means ± SEM for 15 animals per group. *a p* < 0.05 vs. SD; *b p* < 0.05 vs. HFHS.

**Table 2:** Impact of diets on lipid profile, inflammation, and tissue damage in plasma of mice at the end of 12 weeks of protocol.

	SD (*n* = 15)	HFHS (*n* = 15)	HFr (*n* = 15)
Triglycerides (mg/dL)	23.61 ± 1.78	41.60 ± 4.22 *a*	37.30 ± 4.63 *a*
Cholesterol (mg/dL)	85.45 ± 11.94	173.6 ± 10.9 *a*	252.3 ± 27.91 *a*,*b*
IL-6 (pg/mL)	4.36 ± 0.16	11.87 ± 0.34 *a*	9.61 ± 0.86 *a*
LDH (U/L)	230.70 ± 19.73	303.60 ± 25.91 *a*	297.20 ± 25.01 *a*

Data are means ± SEM for 15 animals per group. *a p* < 0.05 vs. SD; *b p* < 0.05 vs. HFHS.

## Data Availability

The raw data supporting the conclusions of this article will be made available by the authors, without undue reservation.

## Supplementary Materials

The following supporting information can be downloaded at: https://www.mdpi.com/article/10.3390/nu15234874/s1, Supplementary Figures file S1: Heart; Supplementary Figures file S2: Tibialis Anterior skeletal muscle.
