# Supplementary material for: Different Effects of High-Fat/High-Sucrose and High-Fructose Diets on Advanced Glycation End-Product Accumulation and on Mitochondrial Involvement in Heart and Skeletal Muscle in Mice
Source: Nutrients. 2023 Nov 22;15(23):4874. doi: 10.3390/nu15234874 (PMC10708161; doi:10.3390/nu15234874)
Supplement: Supplementary file 1 [file nutrients-15-04874-s001.zip › nutrients-2696929-supplementary.pdf]

Supplementary Figures file S1: Heart

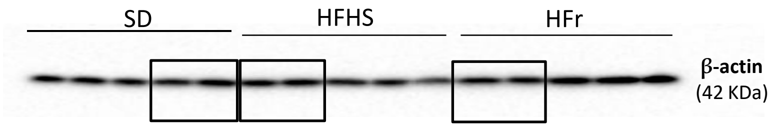

Supplementary Figure S1a. Representative western blotting for β-actin in mouse heart.
Representative whole blots of β-actin protein used in manuscript.
SD (Standard Diet, controls), HFHS (High-Fat High-Sucrose) and HFr (High-Fructose).
The bands enclosed in the boxes are reported in Figure 5.

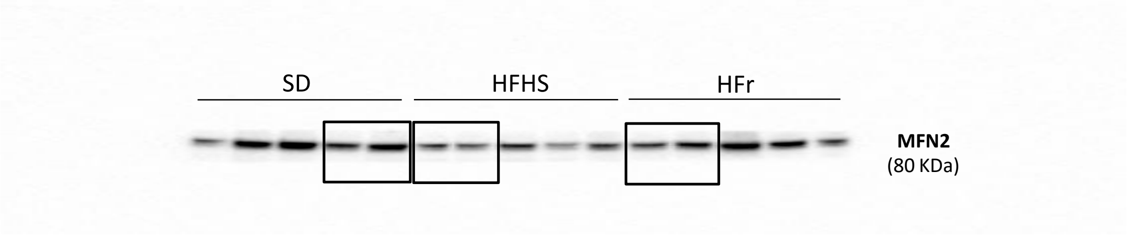

Supplementary Figure S1b. Representative western blotting for MFN2 in mouse heart.
Representative whole blots of MFN2 protein used in manuscript.
SD (Standard Diet, controls), HFHS (High-Fat High-Sucrose) and HFr (High-Fructose).
The bands enclosed in the boxes are reported in Figure 8 (A).

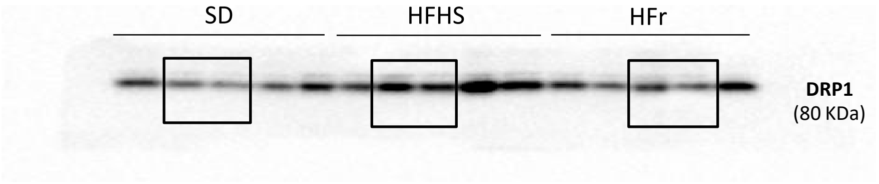

Supplementary Figure S1c. Representative western blotting for DRP1 in mouse heart.
Representative whole blots of DRP1 protein used in manuscript.
SD (Standard Diet, controls), HFHS (High-Fat High-Sucrose) and HFr (High-Fructose).
The bands enclosed in the boxes are reported in Figure 8 (B).

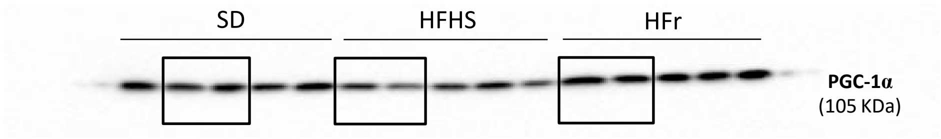

Supplementary Figure S1d. Representative western blotting for PGC-1α in mouse heart.
Representative whole blots of PGC-1α protein used in manuscript.
SD (Standard Diet, controls), HFHS (High-Fat High-Sucrose) and HFr (High-Fructose).
The bands enclosed in the boxes are reported in Figure 7 (A).

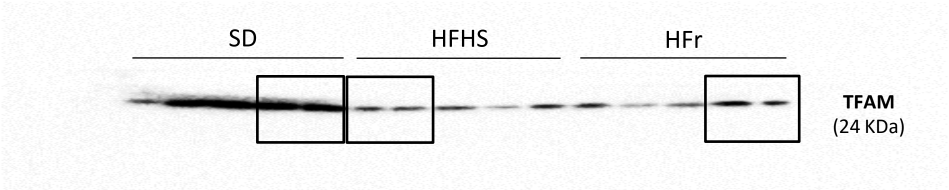

Supplementary Figure S1e. Representative western blotting for TFAM in mouse heart.
Representative whole blots of TFAM protein used in manuscript.
SD (Standard Diet, controls), HFHS (High-Fat High-Sucrose) and HFr (High-Fructose).
The bands enclosed in the boxes are reported in Figure 7 (B).

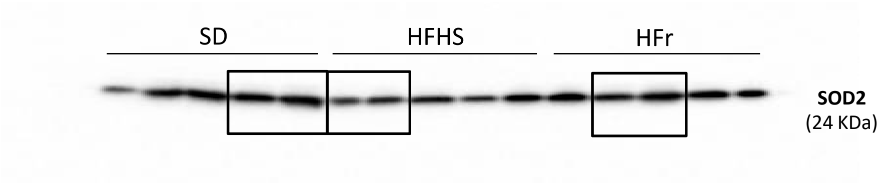

Supplementary Figure S1f. Representative western blotting for SOD2 in mouse heart.
Representative whole blots of SOD2 protein used in manuscript.
SD (Standard Diet, controls), HFHS (High-Fat High-Sucrose) and HFr (High-Fructose).
The bands enclosed in the boxes are reported in Figure 5 (A).

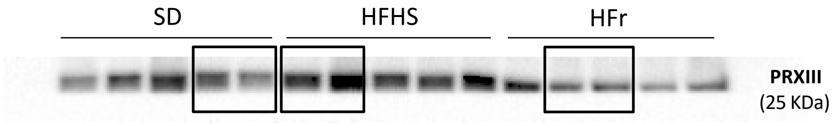

Supplementary Figure S1g. Representative western blotting for PRXIII in mouse heart.
Representative whole blots of PRXIII protein used in manuscript.
SD (Standard Diet, controls), HFHS (High-Fat High-Sucrose) and HFr (High-Fructose).
The bands enclosed in the boxes are reported in Figure 5 (B).

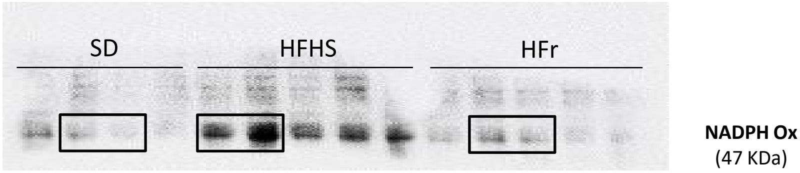

Supplementary Figure S1h. Representative western blotting for NADPH Ox in mouse heart.
Representative whole blots of NADPH Ox protein used in manuscript.
SD (Standard Diet, controls), HFHS (High-Fat High-Sucrose) and HFr (High-Fructose).
The bands enclosed in the boxes are reported in Figure 3 (C).

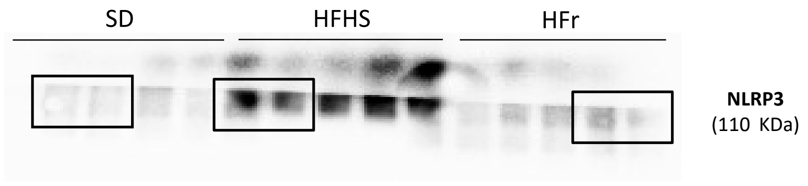

Supplementary Figure S1i. Representative western blotting for NLRP3 in mouse heart.
Representative whole blots of NLRP3 protein used in manuscript.
SD (Standard Diet, controls), HFHS (High-Fat High-Sucrose) and HFr (High-Fructose).
The bands enclosed in the boxes are reported in Figure 3 (A).

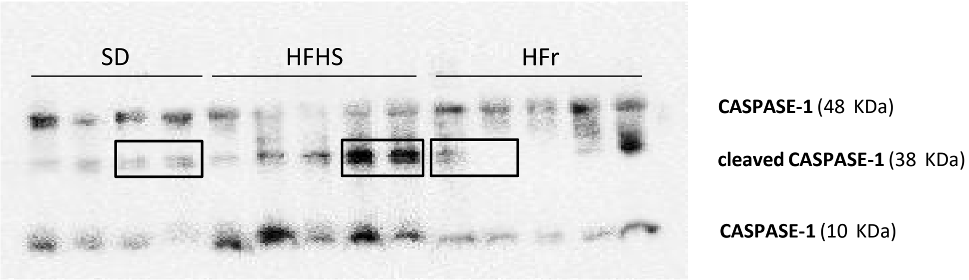

Supplementary Figure S1j. Representative western blotting for CASPASE-1 in mouse heart.
Representative whole blots of cleaved CASPASE-1 protein used in manuscript.
SD (Standard Diet, controls), HFHS (High-Fat High-Sucrose) and HFr (High-Fructose).
The bands enclosed in the boxes are reported in Figure 3 (B).

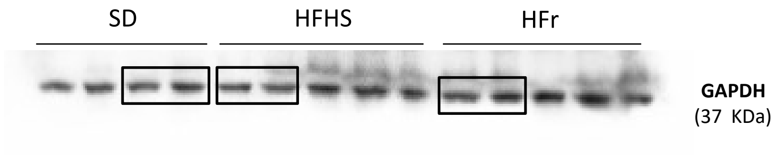

Supplementary Figure S1k. Representative western blotting for GAPDH in mouse heart.
Representative whole blots of GAPDH protein used in manuscript.
SD (Standard Diet, controls), HFHS (High-Fat High-Sucrose) and HFr (High-Fructose).
The bands enclosed in the boxes are reported in Figure 3 (A) and 3 (C).

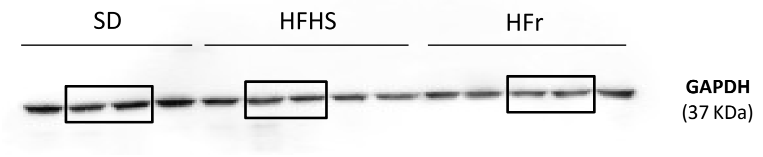

Supplementary Figure S1l. Representative western blotting for GAPDH in mouse heart.
Representative whole blots of GAPDH protein used in manuscript.
SD (Standard Diet, controls), HFHS (High-Fat High-Sucrose) and HFr (High-Fructose).
The bands enclosed in the boxes are reported in Figure 3 (B).

Supplementary Figures file S2: Tibialis anterior skeletal muscle

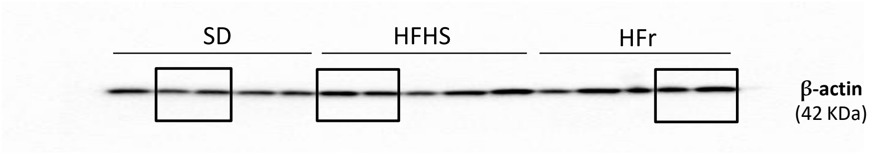

Supplementary Figure S2a. Representative western blotting for β-actin in mouse Tibialis Anterior skeletal muscle.

Representative whole blots of β-actin protein used in manuscript.

SD (Standard Diet, controls), HFHS (High-Fat High-Sucrose) and HFr (High-Fructose).

The bands enclosed in the boxes are reported in Figure 5.

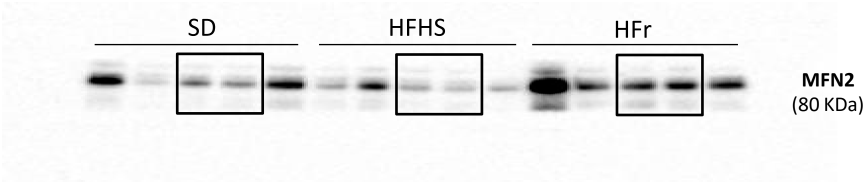

Supplementary Figure S2b. Representative western blotting for MFN2 in mouse Tibialis Anterior skeletal muscle.

Representative whole blots of MFN2 protein used in manuscript.

SD (Standard Diet, controls), HFHS (High-Fat High-Sucrose) and HFr (High-Fructose).

The bands enclosed in the boxes are reported in Figure 8 (A).

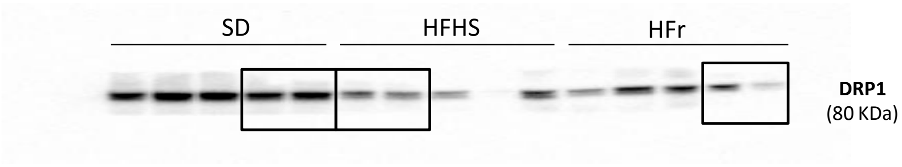

Supplementary Figure S2c. Representative western blotting for DRP1 in mouse Tibialis Anterior skeletal muscle.

Representative whole blots of DRP1 protein used in manuscript.

SD (Standard Diet, controls), HFHS (High-Fat High-Sucrose) and HFr (High-Fructose).

The bands enclosed in the boxes are reported in Figure 8 (B).

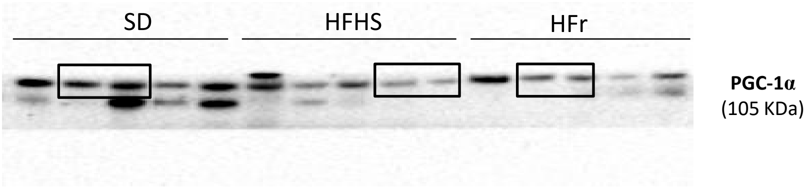

Supplementary Figure S2d. Representative western blotting for PGC-1α in mouse Tibialis Anterior muscle.

Representative whole blots of PGC-1α protein used in manuscript.

SD (Standard Diet, controls), HFHS (High-Fat High-Sucrose) and HFr (High-Fructose).

The bands enclosed in the boxes are reported in Figure 7 (A).

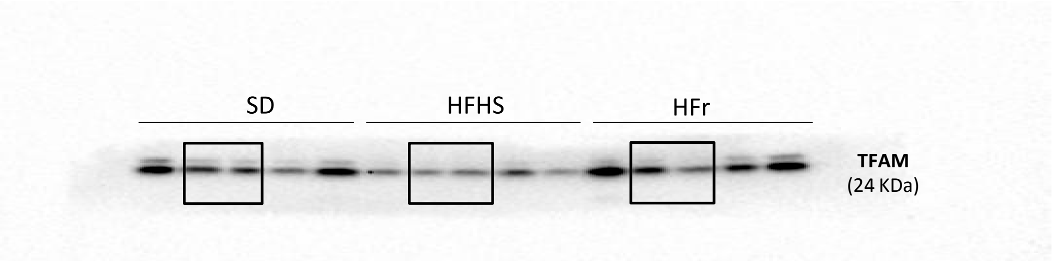

Supplementary Figure S2e. Representative western blotting for TFAM in mouse Tibialis Anterior skeletal muscle.

Representative whole blots of TFAM protein used in manuscript.

SD (Standard Diet, controls), HFHS (High-Fat High-Sucrose) and HFr (High-Fructose).

The bands enclosed in the boxes are reported in Figure 7 (B).

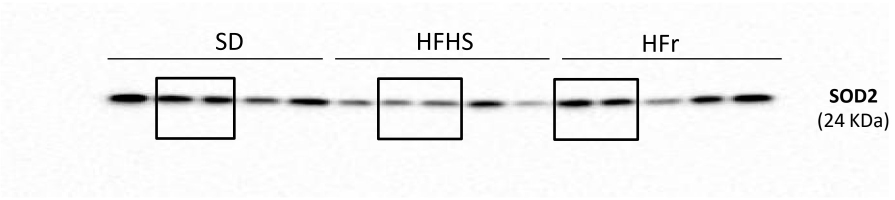

Supplementary Figure S2f. Representative western blotting for SOD2 in mouse Tibialis Anterior skeletal muscle.

Representative whole blots of SOD2 protein used in manuscript.

SD (Standard Diet, controls), HFHS (High-Fat High-Sucrose) and HFr (High-Fructose).

The bands enclosed in the boxes are reported in Figure 5 (A).

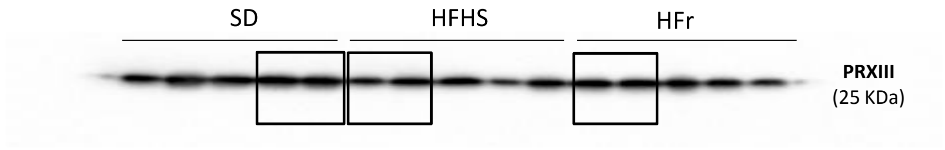

Supplementary Figure S2g. Representative western blotting for PRXIII in mouse Tibialis Anterior skeletal muscle.

Representative whole blots of PRXIII protein used in manuscript.

SD (Standard Diet, controls), HFHS (High-Fat High-Sucrose) and HFr (High-Fructose).

The bands enclosed in the boxes are reported in Figure 5 (B).

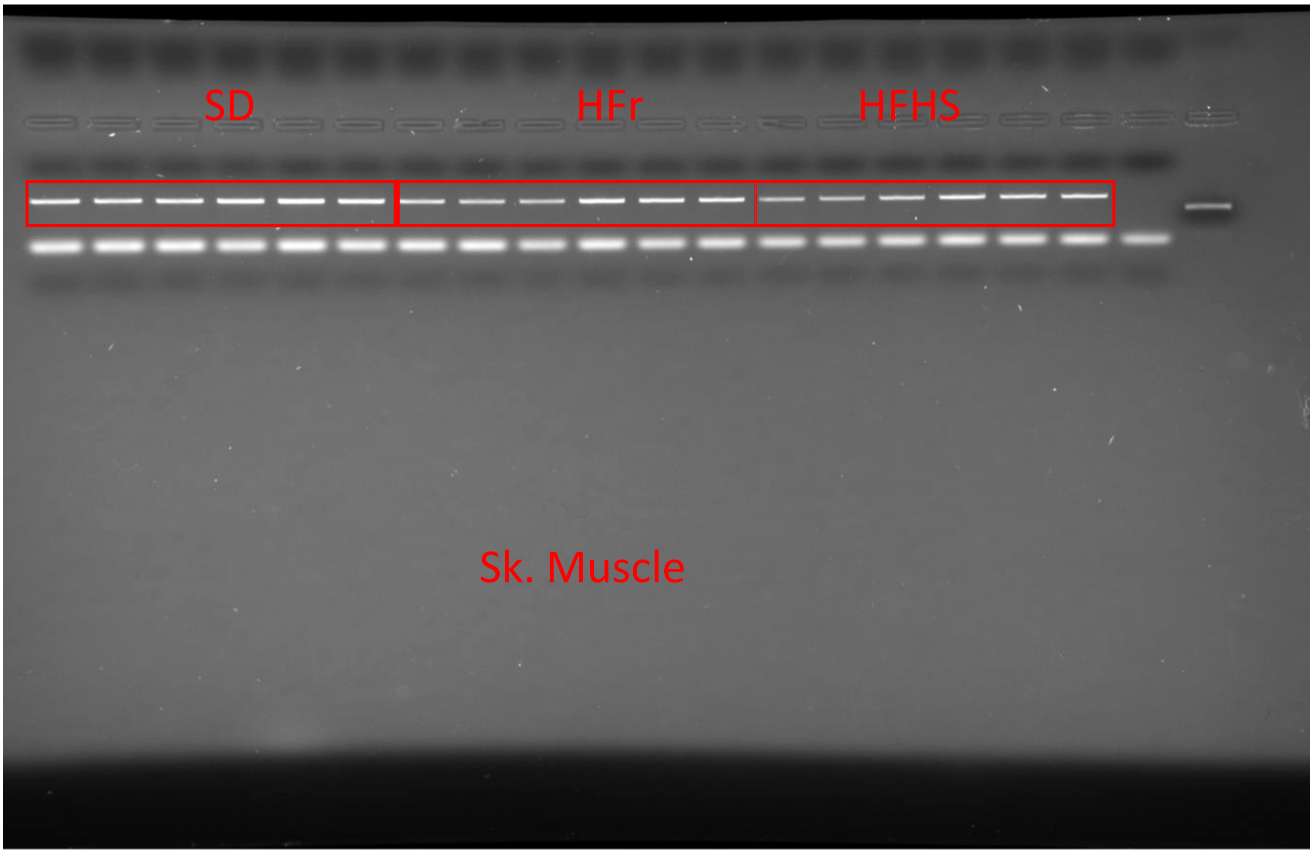

Supplementary Figure S2h. Representative agarose gel for amplicons in mouse TibialisAnterior skeletal muscle.

Representative agarose gel showing amplicons obtained from Fpg-treated and untreated total DNA. SD (Standard Diet, controls), HFHS (High-Fat High-Sucrose) and HFr (High-Fructose).

The bands enclosed in the boxes are reported in Figure 6 (C).

List of Abbreviations

**AGEs**	Advanced Glycation End products
**AUC**	Area Under Curve
**CML**	Nε-(carboxymethyl)-lysine
**CVD**	Cardiovascular Diseases
**DNL**	De novo Lipogenesis
**DRP1**	Dynamin Related Protein 1
**ECL**	Enhanced Chemiluminescence
**ELISA**	Enzyme-Linked Immunosorbent Assay
**F.I.**	Fusion Index
**FPG**	Formamidopyrimidine/8-oxoguanine DNA N-glycosylase
**GAPDH**	Glyceraldehyde-3-phosphate Dehydrogenase
**GOLD**	6-{1-[(5S)-5-ammonio-6-oxido- 6-oxohexyl]imidazolium-3-yl}-L-norleucine/glyoxylderived Lysine Dimer
**HbA1c**	Hemoglobin A1c
**HF**	High Fat
**HFHS**	High Fat High Sucrose diet
**HFr**	High Fructose diet
**HRP**	Horseradish Peroxidase
**IL-1β**	Interleukin-1β
**IL-6**	Interleukin-6
**IL-18**	Interleukin-18
**ITT**	Insulin Tolerance Test
**LDH**	Lactate Dehydrogenase
**MQC**	Mitochondrial Quality Control
**MFN2**	Mitofusin 2
**MOLD**	6-{1-[(5S)-5-ammonio-6-oxido-6-oxohexyl]-4-methyl-imidazoli/methylglyoxal-Lysine Dimer
**MS**	Metabolic Syndrome
**mtDNA**	Mitochondrial DNA
**NADPHox**	Nicotinamide Adenine Dinucleotide Phosphate Hydrogen oxidase
**NAFLD**	Non-alcoholic Fatty Liver Disease
**NLRP3**	Nucleotide-binding domain, Leucine-Rich–containing family, Pyrin domain–containing-3
**OGTT**	Oral Glucose Tolerance Test
**OH8dG**	8-hydroxydeoxyguanosine
**OXPHOS**	Oxidative Phosphorylation
**PGC-1α**	Peroxisome proliferator-activated receptor-Gamma Coactivator 1alpha
**PRXIII**	Peroxiredoxin 3
**PVDF**	Polyvinylidene Difluoride
**qRT-PCR**	Quantitative Real-Time Polymerase Chain Reaction
**RAGE**	Receptor for Advance Glycation End-products
**ROS**	Reactive Oxigen Species
**SD**	Standard Diet
**SDS-PAGE**	Sodium Dodecyl Sulphate - Polyacrylamide Gel Electrophoresis
**SEM**	Standard Error of Mean
**SOD2**	Superoxide Dismutase 2
**T2D**	Type 2 Diabetes
**TA**	Tibialis Anterioris
**TFAM**	Transcription Factor A, mitochondrial
**UHPLC**	Ultra-High-Performance Liquid Chromatography
**WT**	Wild Type
